# Disorders of Puberty in Severely Neurologically Impaired Children: Is Delayed Puberty an Underestimated Problem?

**DOI:** 10.3389/fped.2019.00462

**Published:** 2019-11-14

**Authors:** Valeria Calcaterra, Hellas Cena, Annalisa De Silvestri, Marco Di Mitri, Gloria Pelizzo

**Affiliations:** ^1^Pediatric and Adolescent Unit, Department of Internal Medicine, University of Pavia, Pavia, Italy; ^2^Pediatric Endocrinologic Unit, Department of Maternal and Children's Health, Fondazione IRCCS Policlinico San Matteo, Pavia, Italy; ^3^Clinical Nutrition and Dietetics Service, Unit of Internal Medicine and Endocrinology, ICS Maugeri IRCCS, Pavia, Italy; ^4^Laboratory of Dietetics and Clinical Nutrition, Department of Public Health, Experimental and Forensic Medicine, University of Pavia, Pavia, Italy; ^5^Biometry & Clinical Epidemiology, Scientific Direction, Fondazione IRCCS Policlinico San Matteo, Pavia, Italy; ^6^Pediatric Surgery Department, Children's Hospital “G. Di Cristina, ” ARNAS Civico-Di Cristina-Benfratelli, Palermo, Italy

**Keywords:** children, disability, delayed puberty, malnutrition, adolescents

## Abstract

**Introduction:** In children with disabilities, precocious puberty (PP) has been reported, however there is a paucity of studies on delayed puberty (DP) in neurologically impaired (NI) children.

**Patients and Methods:** We retrospectively evaluated 65 patients with severe disabilities (6–18 years). DP was considered whenever the following criteria where satisfied, respectively, for girls and boys, absence of breast development by age 13 or menarche by age 15, absence of at least 4 mL testicular growth volume or 2.5 cm length by age 14. PP was defined as the presence of puberty signs at <8 and 9 years of age, respectively, for girls and boys. In all patients, a physical examination was performed and a family history of DP was obtained. A hormonal panel was evaluated when puberty disorders were detected. As a control group we evaluated 50 age-matched healthy subjects.

**Results:** Puberty disorders were observed in 12 NI patients and in one control (18.5 vs. 2%, *p* < 0.01). DP was detected in 8 NI subjects (3M/5F) and in one healthy boy (*p* = 0.04), without differences between genders among patients from the NI group (*p* = 0.2), and compared with the controls (*p* = 0.4). In five of the eight NI subjects, Tanner stage 1 was observed; in three subjects adrenarche was present without pubertal progression for more than 2 years. Low levels of gonadotropins were detected in all NI subjects with DP. The number of subjects with a BMI <-3SDS was higher in NI patients with DP compared to NI subjects with normal puberty (*p* < 0.01); normal weight was detected in one healthy boy. The family history for pubertal delay was negative in all NI patients with DP and positive in the control subject.

**Conclusion:** NI children and adolescents may experience delayed pubertal changes. An endocrinological follow-up with pubertal development monitoring is strongly recommended in order to evaluate whether targeted interventions may improve outcomes.

## Introduction

Puberty is a physiologic process marked by the appearance of secondary sex characteristics (1). The absence of sexual maturation signs at 2–2.5 standard deviations or more than the mean age (ca. 13 year in girls and 14 year in boys) was defined as Delayed Puberty (DP) (2).

In children and adolescents with disabilities, the pattern of sexual maturation may differ from that of children in the general population (3) with possible abnormal puberty development. Brain injury during structural and functional brain maturation in the pre-, peri-, or post-natal period may negatively impact the development of pubertal changes (4).

Sexual maturation may also be delayed; (3, 5) moreover, there is a paucity of studies on pubertal delay in neurologically impaired (NI) children. For this reason, in this report, we focused our attention on the occurrence of DP in NI children and adolescents in order to define the need for appropriate surveillance to prevent adverse effects related to delayed pubertal timing.

## Patients and Methods

### Patients

We retrospectively evaluated 65 patients (37M/28F) with severe disabilities aged 6–18 years (mean age 13.5 ± 0.4 years), referred to our Endocrinological Unit for auxological evaluation or Pediatric Surgical Unit for treatment and/or management of nutrition support. All of the patients, were bedridden, whether they lived at home or in sheltered communities. Diagnoses included cerebral palsy due to hypoxic ischemic damage (44%), severe psychomotor delay in dysmorphic syndromes without a clear association with pubertal disorders (30%), epileptic encephalopathy (26%). Anticonvulsive drugs (at least two of the following: phenobarbital, valproic acid, phentoyn, lamotrigine, topiramate, carbamazepine, and clonazepam) were reported in 58/65 of the patients (89.2%).

As a control group we evaluated 50 age-matched healthy subjects (21F/29M; mean age 12.0 ± 0.5 years) referred by their general practitioner or primary care pediatrician to our Outpatient Clinic for auxological evaluation. The study was approved by the Fondazione IRCCS Policlinico San Matteo Institutional Review Board and according to the 1964 Declaration of Helsinki. After receiving information on the nature of the study, written informed consent was obtained from the parents of the participating children.

### Methods

All patients underwent a physical examination including: weight, height, body segment measurements, as previously described (6). Body Mass Index (BMI) was calculated by dividing the patient's weight in kilograms by the square of the height in meters and the BMI Z-score (standard deviation), was calculated using UptoDate calculators based on the Centers for Disease Control and Prevention growth charts (7, 8).

Pubertal development was classified according to Marshall and Tanner (9, 10) and the population was classified as stage one corresponding to Tanner 1; stage 2 corresponding to Tanner 2–3; and stage 3 corresponding to Tanner 4–5. The absence of breast development by age 13 or menarche by age 15, absence of at least 4 mL testicular growth volume, or 2.5 cm length by age 14 were considered delayed puberty (DP), respectively, for girls and boys (2, 9, 10). Precocious puberty was defined as the presence of puberty signs before 8 and 9 years of age, respectively, for girls and boys (9, 10). An extended family history of pubertal delay was recorded and patients were classified as having at least a familiar tendency to DP (men with pubertal onset >13 years and/or growth spurt >15 years; women with menarche >14 years) or diagnosed with DP (men with pubertal onset >14 years and/or growth spurt >16 years; women with menarche >15 years) (2). Gonadotropin levels were evaluated when disorders of puberty were detected and LH, FSH were measured with a chemiluminescent immunometric assay.

### Statistical Analysis

Continuous variables are presented as the mean ± standard deviation; categorical variables are expressed as numbers or percentage of patients. The statistical significance of the continuous variable comparisons was assessed using the unpaired Student's *t*-test; the comparison of categorical variables was conducted using the chi square test or Fisher's Exact test if there was a small (<5) expected cell size. Stata 15.1 (StataCorp, College Station, Tex., USA) was used for computation. All statistical tests were two-tailed and a *p* < 0.05 was considered statistically significant.

## Results

The clinical features of the NI subjects are reported in Table 1. The average age of the disabled children at evaluation was 12.7 ± 4.2 years for males and 14.6 ± 3.4 years for females, without significant differences with the controls (*p* = 0.09).

**Table 1:** Clinical features of the NI subjects.

	**All NI subjects (*n* = 65)**	**NI subjects with DP (*n* = 8)**	**NI subjects without DP (*n* = 57)**	p^*^
Sex (M/F)	37/28	3/5	34/23	0.2
Age at evaluation	13.5 ± 4.0	14.9 ± 2.6	15.0 ± 3.7	0.9
Diagnosis				
– Hypoxic ischemic damage	28	4	24	
– Epileptic encephalopathy	20	1	19	0.5
– Dysmorphic syndromes	17	3	14	
Pubertal stages				
– Tanner 1	18	5	13	
– Tanner 2-3	15	3	12	<0.01
– Tanner 4-5	32	0	32	
Nutritional support				
– Enteral feeding	60	7	53	0.6
– Oral feeding	5	1	4	
Anticovulsivant drug	58	7	51	0.8
Severe malnutrition (BMI<-3SDS)	23	6	17	<0.01
Family history of pubertal delay	1	0	1	0.3

* *NI subjects with DP vs. NI subjects without DP*.

Puberty disorders were observed in 12 NI patients and in one control (18.5 vs. 2%, *p* < 0.01). DP was detected in 8 NI subjects (three males and five females, Table 1) and in one healthy boy (*p* = 0.04), without differences between genders among patients from the NI group (*p* = 0.2), and compared with the controls (*p* = 0.4). In four NI patients (6.1%), PP was also detected (two central precocious, treated with GnRH analog and 2 premature adrenarche not treated).

The average age of the NI subjects diagnosed with DP was 12.6 ± 4.4 years for males and 13.9 ± 2.7 years for females. As reported in Table 1, in five of the eight NI subjects, Tanner stage 1 was observed (mean age 13.8 ± 3.8 years); in three subjects adrenarche was present (three patients with PH 2 and 1 with PH3) without pubertal progression for more than 2 years.

Low levels of gonadotropins were detected in all NI subjects with DP (Girls: LH 0.7 ± 0.5 IU/liter; FSH 1.4 ± 1.2 IU/liter. Boys: LH 0.4 ± 0.3 IU/liter; FSH 0.8 ± 1.0 IU/liter). Magnetic resonance imaging was performed in only one patient with DP; no hypothalamic-pituitary malformations were observed.

The number of subjects with a BMI <-3SDS (severely underweight) (11, 12), was higher in NI patients with DP compared to NI subjects with normal puberty (*p* < 0.01); no association between DP and type of nutritional support (*p* = 0.6) was noted, Table 1. Normal weight was detected in one healthy boy.

Seven NI patients diagnosed with DP were treated with at least two anticonvulsive drugs (*p* = 0.8, Table 1).

The family history for pubertal delay was negative in all NI patients with DP (Table 1) and positive in the control subject.

## Discussion

Puberty onset for individuals living in relatively similar conditions, is characterized by a 4–5 years physiological variation in sexual maturation (1–3), and is expected to occur between 8–13 and 9–14 years of age, respectively, in girls and boys (2).

NI children could experience disorders of puberty in term of precocious or delayed puberty. Pediatric patients affected by neurodevelopmental disabilities are 20 times more at risk of premature pubertal changes compared to the general population (4). Many insults to the central nervous system including birth asphyxia, congenital malformation and infections can cause PP (4). Severe brain damage and use of antiepileptic medication may affect a number of neurotransmitter pathways involved in gonadotropin control, inducing an activation of the hypothalamic-pituitary-gonadal axis (13). Additionally, precocious sexual development has been described in several genetic disorders and malformative syndromes with midline brain involvement (4).

In this brief report, we documented a high occurrence of DP in NI children and adolescents, without differences between genders and in the absence of a family history.

The etiology of delayed puberty in NI patients may be multifactorial (1, 2, 14), but in our small study population a causative factor was not identified. However, it is known that secondary chronic malnutrition reported in these patients (6) largely contributes to their delayed initiation of pubertal development. Nutrition and nutritional status impact pubertal development (9), and are responsible for up to 25% of the changes in puberty timing. Adolescent sexual development, both in boys and girls, is affected by chronic malnutrition during childhood (14). NI patients suffering from secondary malnutrition (6) may also suffer delayed puberty onset.

Despite BMI is not a reliable indicator of nutritional status and it may not have an etiological role in pubertal disorders (4), in this pediatric population severe undernutrition is associated with body composition disturbances (15). Undernutrition and low body fat mass, or an altered ratio of lean mass to body fat, seem to play a crucial role in the initiation of puberty (16).

Other explanations for puberty delay may be associated with cumulative stress (17) frequently reported in this fragile population; metabolic and physiologic stress activate hypothalamic-pituitary-adrenal axis and suppress hypothalamic-pituitary-gonadal axis (18). Additionally the use of medications could influence the levels of different sex hormones resulting in effects on the pubertal development (4).

Brain malformation associated with pituitary dysfunction can also be considered (19). However, the role of genetic factors and fetal programming should not be excluded (1–3).

Although not always acknowledged, self-limiting DP is not totally benign and could be related to an increased risk for metabolic and cardiovascular disorders, bone mineral density modifications as well as fracture risk later on in life. This is observed particularly in disabled pediatric patients, in whom early cardio-vascular and metabolic impairment is already present due to multisystem dysregulation (20).

We recognize that there are some limitations to this study starting with the relatively small sample size that was due to difficulties in enrolling and studying neuro-cognitively disabled children. Secondly, due to the severity of the clinical condition, MRI was performed in only one patient with DP. Therefore, the presence of hypothalamic-pituitary malformations cannot be excluded in the other patients. Additionally, we considered a cohort of children with a high grade of disability that can be implicated in severe biological dysregulation influencing pubertal progression. A comparison with a group with moderate disabilities would be useful in future studies. Finally, long-term anticonvulsant drugs may be associated with sexual maturation disorders; all of the patients received polytherapy and the effects of single anticonvulsants were not pondered, however since all the subjects were on anticonvulsant medications this can be considered a systematic error. Despite the acknowledged limitations, which do not allow generalizations, the present report highlights that NI children and adolescents also experience delayed pubertal changes. Further research is recommended to establish whether DP is a primary manifestation or a secondary consequence of chronic impaired health and malnutrition.

In conclusion, NI children and adolescents may experience delayed pubertal changes. Once accessed the care process all patients should undergo an endocrinological assessment and subsequently evaluated twice a year to detect precocious or delayed pubertal development in order to be cared.

Moreover, an endocrine monitoring should be also required at least once a year after pubertal development, to evaluate whether targeted interventions can improve outcomes, as well as reassessed form a nutritional point of view to offer tailored nutritional interventions and limit malnutrition comorbidities (21, 22).

## Data Availability Statement

The datasets generated for this study are available on request to the corresponding author.

## Ethics Statement

This study involving human participants is reviewed and approved by Fondazione IRCCS Policlinico San Matteo, Pavia (protocol n.20160001270 for patients and n.20150005231 for historical healthy subjects) and ARNAS Civico-Di Cristina-Benfratelli (register n. 354 Civico 2016). Written informed consent to participate in this study was provided by the participants' legal guardian/next of kin in accordance with the Declaration of Helsinki.

## Author Contributions

VC, GP, and HC contributed to the study concept design, drafted, and revised the article. MD contributed to the acquisition of data and drafted the article. AD performed the statistical analysis. All of the authors approved the final version.

### Conflict of Interest

The authors declare that the research was conducted in the absence of any commercial or financial relationships that could be construed as a potential conflict of interest.
